# A comparison of methods for analysing compositional data with fixed and variable totals: a simulation study using the examples of time-use and dietary data

**DOI:** 10.1186/s12874-025-02509-1

**Published:** 2025-04-17

**Authors:** Georgia D. Tomova, Rosemary Walmsley, Laurie Berrie, Michelle A. Morris, Peter W. G. Tennant

**Affiliations:** 1 The Alan Turing Institute, British Library, 96 Euston Road, London, NW1 2DB UK; 2 Leeds Institute for Data Analytics, University of Leeds, Leeds, LS2 9NL UK; 3 School of Medicine, University of Leeds, Leeds, LS2 9NL UK; 4 Independent researcher, Penarth, UK; 5 Institute of Geography, School of Geosciences, University of Edinburgh, Edinburgh, EH8 9XP UK; 6 School of Food Science & Nutrition, University of Leeds, Leeds, LS2 9JT UK

**Keywords:** Compositional data, Compositional data analysis, Isotemporal model, Ratio variables, Leave-one-out model, Physical activity, Dietary intake

## Abstract

**Background:**

Compositional data comprise the parts of a ‘whole’ (or ‘total’), which sum to that ‘whole’. The ‘whole’ may vary between units of analyses, or it may be fixed (constant). For example, total energy intake (a variable total) is the sum of intake from all foods or macronutrients. Total time in a day (a fixed total) is the sum of time spent engaging in various activities. There exist different approaches to analysing compositional data, such as the isocaloric or isotemporal model, ratio variables, and compositional data analysis (CoDA). Although the performance of the different approaches has been compared previously, this has only been conducted in real data. Since the true relationships are unknown in real data, it is difficult to compare model performance in estimating a known effect. We use data simulations of different parametric relationships, to explore and demonstrate the performance of each approach under various possible conditions.

**Methods:**

We simulated physical activity time-use and dietary data as examples of compositional data with fixed and variable totals, respectively, using different parametric relationships between the compositional components and the outcome (fasting plasma glucose): linear, log_2_, and isometric log-ratios. We evaluated the performance of a range of generalised linear and additive models as well as CoDA, in estimating a 1-unit and either 10-unit (for physical activity) or 100-unit (for dietary data) reallocations under each parametric scenario. We simulated 10,000 datasets with 1,000 observations in each.

**Results:**

The performance of each approach to analysing compositional data depends on how closely its parameterisation matches the true data generating process. Overall, we demonstrated that the consequences of using an incorrect parameterisation (e.g. using CoDA when the true relationship is linear) are more severe for larger reallocations (e.g. 10-min or 100-kcal) than for 1-unit reallocations. The implications of choosing an unsuitable approach may be starker in compositional data with variable totals. For example, while models with ratio variables are mathematically equivalent to linear models in compositional data with fixed totals, their estimates may be radically different for variable totals.

**Conclusions:**

Compositional data with fixed and variable totals behave differently. All existing approaches to analysing such data have utility but need to be carefully selected. Investigators should explore the shape of the relationships between the compositional components and the outcome and chose an approach that matches it best.

**Supplementary Information:**

The online version contains supplementary material available at 10.1186/s12874-025-02509-1.

## Background

It is widely understood that physical activity and a healthy diet are good for short- and long-term health [1–3], and may have a positive impact on planetary health [4]. Measuring and analysing these data remains challenging. In part, this is due to their compositional nature. Compositional data comprise the parts of a ‘whole’ (or ‘total’), which together sum up to that ‘whole’ [5, 6]. For example, total energy intake is the sum of intake from all foods or macronutrients and total time in a day is the sum of time spent engaging in various activities. Depending on the context, the ‘total’ may be the same across the units of analysis (i.e. be *fixed* or *constant*), such as 24 h in a day for a physical activity diary, or it may vary (i.e. be *variable*) such as daily energy intake.

To generate meaningful insights from diet and physical activity research, the compositional nature of these data and the nuances of analysing such data must be considered. Compositional data have been discussed widely in the past, most notably (and extensively) by John Aitchison. Following Karl Pearson’s warnings about ‘spurious correlations’ in the analyses of ratio variables and compositional data [7], Aitchison developed the modern theory of compositional data around geometrical principles and established the field of compositional data analysis (CoDA) ( [5, 8, 9] among others). Originally exemplified in geochemical data [5], it is now recognised that there exist many scenarios in which data can be conceptualised as being compositional by breaking down a variable into smaller components or by aggregating similar variables [6].

Recently, compositional data have been examined from a causal inference perspective using causal directed acyclic graphs (DAGs) [6, 10]. Given the intuitive nature of DAGs, this approach has the potential to introduce compositional data to a wider audience. The previous and more traditional descriptions relied upon substantial understanding of geometric theory which risks making them inaccessible to applied researchers, though there exist attempts to introduce these concepts in a more accessible way [11, 12].

However, since DAGs are non-parametric, they provide limited guidance on some of the parametric challenges with analysing compositional data, such as whether and when it is sufficient to use simple linear models. There are three main parametric approaches for the analysis of compositional data, the utilisation of which differs substantially between fields: the isocaloric and isotemporal models (also known as ‘leave-one-out’ models), models using ratio (or proportion) variables, and models using log-ratio transformations.

### The ‘leave-one-out’ isocaloric and isotemporal models

Suppose we are interested in the effect of a particular exposure variable instead of a specified alternative (e.g. consuming more unsaturated fat instead of saturated fat, or spending more time gardening instead of reading books). This effect may be estimated in an experimental (intervention) study by substituting the main exposure for the specified alternative, while keeping all other nutrients or activities unchanged.

Alternatively, this may be estimated statistically using a regression model of the following type:

$$Y=a_0+a_1x_1+a_2x_2+\dots+a_{n-1}x_{n-1}\left(+a_nx_{total}\right)+e,$$

where $Y$ is the outcome (e.g. fasting plasma glucose), ${x}_{1},\dots , {x}_{n}$ are compositional components (e.g. carbohydrates, fat, and protein), ${x}_{total}$ is the compositional total (e.g. total energy intake due to carbohydrates, fat, and protein) such that ${x}_{total}={x}_{1}+\dots +{x}_{n}.$ Note: ${x}_{total}$ is implicit when the total is fixed, and cannot be included in the model, but must be included whenever the total varies. At least one term (e.g. ${x}_{n}$ or protein) must be ‘left out’ of the model (hence the name), and this acts as the reference level for the substitution. The coefficient for the exposure of interest, e.g. ${a}_{1}$ for ${x}_{1}$ (e.g. carbohydrates) represents the effect exerted on the outcome $Y$ by substituting 1 unit of ${x}_{1}$ for ${x}_{n}$ (carbohydrates for protein). Since the resulting substitutions relate to the same number of calories (or the same amount of time, for example), these models are often described as ‘isocaloric’ or ‘isotemporal’, respectively [13, 14].

Alternatively, where the total varies, the same effect may be estimated using a similar model which includes all compositional components (also known as a ‘partition’ or ‘decomposition’ model) of the following type.

$$Y=b_0+b_1x_1+b_2x_2+\dots+b_{n-1}x_{n-1}+b_nx_n+e,$$

where the derived effect may be obtained by subtracting the coefficients for the exposure (e.g. ${b}_{1}$ for ${x}_{1}$) from the specified alternative (${b}_{n}$ for ${x}_{n}$) [15–17].

### Ratio variables

Since compositional components are part of a ‘total’, they can also be considered in terms of proportions or ratios of that total. This may be of interest when it is believed that the proportion of the total explained by a particular component is more meaningful than its absolute amount. The use of ratio variables for compositional data are particularly common in nutritional epidemiology, where this approach is referred to as the ‘nutrient density model’ [13, 18]. Typically, this is conducted using a linear regression model of the type:

$$Y= {c}_{0}+{c}_{1}\frac{{x}_{1}}{{x}_{total}}+{c}_{2}\frac{{x}_{2}}{{x}_{total}}+\dots \left(+{c}_{n}\frac{{x}_{n}}{{x}_{total}}\right)+e,$$

where $Y$ is the outcome, ${x}_{1},\dots , {x}_{n}$ are compositional components, and ${x}_{total}$ is the compositional total such that ${x}_{total}={x}_{1}+\dots +{x}_{n}.$ Where the total is fixed, at least one term must be ‘left out’, which acts as the reference. Where the total varies, an alternative version of this approach (known in nutritional epidemiology as the ‘multivariable nutrient density model’) additionally involves adjustment for the total, ${x}_{total}$, by including it as a covariate in the model.

Naïvely, one might expect the interpretation of ${c}_{1}$ to be the same as ${a}_{1}$ above, albeit rescaled into percentage or proportion units. In practice, where the total varies (and is not additionally conditioned), this approach may produce misleading results [17].

### Compositional Data Analysis (CoDA)

The field of compositional data analysis (CoDA) primarily builds on the work of its founder, John Aitchison. CoDA is a framework of methods for analysing compositional data that rests on the fact that compositional components fall on a constrained geometric space, known as a simplex, rather than the unconstrained Euclidean (real) space. This constraint always applies to compositional data with a fixed total. However, when the total varies, we can choose to move between the Euclidean space and the simplex by ‘closing’ the data with a suitable transformation, such as dividing by, or conditioning on, the total. When the compositional data are closed, the components can only ever be analysed as relative to each other [5], and these relative relationships are the focus of CoDA.

To model the compositional components relative to each other, CoDA proposes using log-ratio transformations, such as isometric log-ratio transformations [11] that are applied to the compositional components in a model of the following type:

$$Y= {d}_{0}+{d}_{1}\text{ln}\left(\frac{{x}_{1}}{\sqrt[3]{{x}_{2}\times {x}_{3}\times {x}_{4})}}\right)+ {d}_{2}\text{ln}\left(\frac{{x}_{2}}{\sqrt[2]{{x}_{3}\times {x}_{4})}}\right)+ {d}_{3}\text{ln}\left(\frac{{x}_{3}}{{x}_{4}}\right)+ e$$

where $Y$ is the outcome, and ${x}_{1}, {x}_{2}, {x}_{3}, {x}_{4}$ are compositional components in a scenario where ${x}_{total}={x}_{1}+\dots +{x}_{4}.$ This ensures that the constrained space of possible values is respected in a similar way that, for example, a logit transformation prevents impossible zero values. The downside is that the interpretation is not as straightforward as with simpler linear regression approaches. Indeed, the coefficients for models containing isometric log-ratio transformations (i.e. ${d}_{1}$, ${d}_{2}$, ${d}_{3}$) are essentially uninterpretable. Interpretable effects may be obtained by comparing predictions of the outcome at different levels of the components [19], although other approaches, such as populating a change-matrix have also been suggested [20]. In health and medicine, CoDA is most commonly used in time-use epidemiology, typically in the context of physical activity, for example for estimating the effect of reallocating different activity behaviours on adiposity, risk of depression, and other health outcomes [21–25]. However, the CoDA approach has also been advocated in the context of nutritional epidemiology and exemplified in studies exploring the effect of various nutrient reallocations on waist circumference or Metabolic Syndrome, for instance [26–28].

### Aim

Despite extensive theoretical literature on the topic, there remains little practical advice on the relative merits and performance of the different approaches to estimating the causal effects of a compositional exposure. Although these approaches have been compared in the past [21, 29, 30], this has only been conducted in real data. Unlike in simulated data, the true data generating process (the process which leads to the observed data distributions), and therefore the true effects, are never known in real data. This makes it difficult to understand some of the general implications of each approach when there is no empirical truth as a benchmark. Therefore, in the study presented here, we use data simulations of different parametric relationships, to explore and demonstrate the performance of each approach under various possible conditions.

## Methods

### Illustrative example

To examine and compare the performance of different approaches to analysing compositional data, we use the examples of diet and physical activity data. Physical activity time-use data are by far the most common example of compositional data where the total is fixed (typically 24 h). Nutritional data, on the other hand, offer an example of compositional data with variable totals. In both fields, all of the above approaches have been used and/or recommended [11, 27]. For simplicity, we use the same illustrative outcome for both settings, fasting plasma glucose (FPG), which is a plausible outcome of interest in either scenario. We simulate a physical activity dataset and a nutrition dataset, in which the causal relationships between all compositional components and the outcome are known, which we refer to as the ‘ground truth’ effects. We evaluate the performance of various analytical models when applied to data that has been simulated to have the following types of relationships between the compositional components and the outcome; 1) simple linear, 2) simple non-linear (log_2_), and 3) non-linear (isometric log-ratios).

### Data simulation

All simulations were conducted using the DagSim package in Python 3.7.3 [31]. DagSim is a framework for simulating data based on a known causal structure such as a DAG, that allows full flexibility in terms of variable types and functional relationships. We conducted separate simulations for compositional data with variable or fixed totals as different steps were required to achieve the necessary relationships among the compositional components. We simulated 10,000 datasets with 1,000 observations in each. Although ubiquitous and critical to any applied analysis, we did not simulate confounding or measurement error because introducing additional complexity may distract from the key messages at hand.

### Fixed totals (physical activity)

The simulation of compositional data with fixed totals has special considerations because all components are jointly dependent on each other. We simulated this dependence by starting with a single time-use component and iteratively deriving each additional component from: 1) the time spent in each previous state, 2) the target proportion of the remaining unallocated time, and 3) noise, drawn from a normal distribution with constant variance. We simulated four physical activity variables with the following target means: sleep (564 min/day), sedentary behaviour (SB) (541 min/day), light physical activity (LPA) (313 min/day), and moderate-to-vigorous physical activity (MVPA) (22 min/day). The target means and variances were chosen to balance the plausibility of the data and the practical requirements of the simulation (e.g. that all values must be positive). The total time across all datasets is constant and equal to 1440 min (24 h)/day. For illustrative purposes, the effect we seek to estimate is the joint (substitution) effect of increasing MVPA and decreasing LPA by equal amounts.

### Variable totals (energy intake)

To simulate compositional data with variable totals, we implemented an approach used previously [17, 32]. Unlike fixed totals, variable compositional totals may differ within and between individuals. A change in one compositional component, therefore, does not necessarily require changes in the values of the other components (unless imposed by experimental or statistical intervention). To illustrate this scenario, we simulated the intake of four macronutrient variables with the following target means: 1) carbohydrates (1000 kcal/day), 2) fat (600 kcal/day), 3) protein (300 kcal/day), and 4) alcohol (100 kcal/day). Total energy intake was subsequently derived from the sum of all macronutrient intakes (in kilocalories), with a resulting mean of 2000 kcal/day. For illustrative purposes, the effect we seek to estimate is the joint (substitution) effect of increasing carbohydrate intake and decreasing protein intake by equal amounts.

### Outcome

To simulate the outcome, FPG, we defined three ground truth scenarios: one where the outcome was linearly determined by the components, one where it was determined via log_2_ relationships, and one where it was determined via isometric log-ratio (ILR) relationships. The log_2_ parameterisation was chosen for the non-linear data-generating scenario because a log relationship was deemed most plausible. The beneficial impacts of physical activity on health outcomes, including diabetes, tend to occur at the lower levels of activity with diminishing returns at the higher levels [33]. Parametrically, this is best represented via a log relationship. In a log relationship, the effect of increasing the exposure indeed diminishes, so a larger effect is observed at the beginning of the scale (e.g., increasing physical activity from 0 min/day to 10 min/day) than for higher values (e.g., increasing from 40 min/day to 50 min/day). ILRs were chosen due to their particular prominence in time-use epidemiology [24].

Model coefficients were selected to 1) provide realistic causal effects for each component, 2) return a mean FPG of 5.5 mmol/l, and 3) so that the target substitution effect for a single unit substitution (i.e. 1 min increase in MVPA and 1 min decrease in LPA, or 1 kcal increase from carbohydrates and 1 kcal decrease from protein) was the same regardless of the specific parametric relationship. The correct coefficients required to obtain the same substitution effect across models with different parametric relationships were calculated by solving simultaneous equations. For larger-unit changes, this equivalence between models is not expected due to the different shape of the relationships. Table 1 contains the true data generating models and ground truth effects for each scenario.

**Table 1:** True data generating models and ground truth effects used in the simulations

True relationship	True data generating model	Ground truth effects	
**Compositional data with a fixed total (physical activity)**		**MVPA instead of LPA**	
		1 kcal	100 kcal
Linear	$FPG= {\alpha }_{0}+{\alpha }_{1}MVPA+ {\alpha }_{2}SB+{\alpha }_{3}Sleep+\varepsilon$, where $\varepsilon \sim N\left(0, {{\sigma }_{1}}^{2}\right)$	−0.020 mmol/l	−0.200 mmol/l
Log_2_	$FPG= {\gamma }_{0}+{\gamma }_{1}{log}_{2}MVPA+ {\gamma }_{2}{log}_{2}SB+{\gamma }_{3}{log}_{2}Sleep+\varepsilon$, where $\varepsilon \sim N\left(0, {{\sigma }_{1}}^{2}\right)$	−0.020 mmol/l	−0.166 mmol/l
ILR	$FPG= {\zeta }_{0}+{\zeta }_{1}\text{ln}\left(\frac{MVPA}{\sqrt[3]{SB\times Sleep\times LPA)}}\right)+ {\zeta }_{2}\text{ln}\left(\frac{SB}{\sqrt[2]{Sleep\times LPA)}}\right)+ {\zeta }_{3}\text{ln}\left(\frac{Sleep}{LPA}\right)+\varepsilon$, where $\varepsilon \sim N\left(0, {{\sigma }_{1}}^{2}\right)$	−0.020 mmol/l	−0.157 mmol/l
**Compositional data with variable totals (energy intake)**		**Carbohydrates instead of protein**	
		1 kcal	100 kcal
Linear	$FPG= {\eta }_{0}+{\eta }_{1}Carbs+ {\eta }_{2}Fat+{\eta }_{3}Alcohol +{\eta }_{4}Total+\varepsilon$, where $\varepsilon \sim N\left(0, {{\sigma }_{2}}^{2}\right)$	0.004 mmol/l	0.400 mmol/l
Log_2_	$FPG= {\lambda }_{0}+{\lambda }_{1}{log}_{2}Carbs+{\lambda }_{2}{log}_{2}Fat+{\lambda }_{3}{log}_{2}Alcohol +{\lambda }_{4}{log}_{2}Protein+\varepsilon$, where $\varepsilon \sim N\left(0, {{\sigma }_{2}}^{2}\right)$	0.004 mmol/l	0.284 mmol/l
ILR	$FPG= {\xi }_{0}+{\xi }_{1}\text{ln}\left(\frac{Carbs}{\sqrt[3]{Fat\times Alcohol\times Protein)}}\right)+ {\xi }_{2}\text{ln}\left(\frac{Fat}{\sqrt[2]{Alcohol\times Protein)}}\right)+{\xi }_{3}\text{ln}\left(\frac{Alcohol}{Protein}\right)+\varepsilon$, where $\varepsilon \sim N\left(0, {{\sigma }_{2}}^{2}\right)$	0.004 mmol/l	0.510 mmol/l

*ILR* isometric log-ratio, *LPA* light physical activity, *MVPA* moderate-or-vigorous physical activity, *SB* sedentary behaviour. Parameter values can be found in Table S2

Full details of the parameters used to derive all variables, as well as other details of the data generation process, are available in the annotated code in Supplementary Code and Supplementary Table 1. The means and standard deviations of all simulated variables are also presented in Supplementary Table 2.

### Evaluation of modelling approaches

All further statistical analyses were conducted in R 4.3.0. For each simulated version of the outcome, we estimated the causal effects of interest using a range of analytical approaches and compared the results to the known ground truth. Table 2 contains all analytical models that were considered in the analyses.

**Table 2:** Analytical models used to estimate the substitution effects of interest

Model number	Model name	Model formula
**Compositional data with a fixed total (physical activity)**		
1A	Linear leave-one-out model (classic “isotemporal model”)	$FPG= {\widehat{\alpha }}_{1}+{\widehat{\alpha }}_{1}MVPA+ {\widehat{\alpha }}_{2}SB+{\widehat{\alpha }}_{3}Sleep+\varepsilon$
1B	Ratio leave-one-out model	$FPG= {\widehat{\beta }}_{0}+{\widehat{\beta }}_{1}\frac{MVPA}{Total}+ {\widehat{\beta }}_{2}\frac{SB}{Total}+{\widehat{\beta }}_{3}\frac{Sleep}{Total}+\varepsilon$
1C	Log2 leave-one-out model	$FPG= {\widehat{\gamma }}_{0}+{\widehat{\gamma }}_{1}{log}_{2}MVPA+ {\widehat{\gamma }}_{2}{log}_{2}SB+{\widehat{\gamma }}_{3}{log}_{2}Sleep+\varepsilon$
1D	GAM leave-one-out model	$FPG= {\widehat{\delta }}_{0}+{f}_{1}\left(MVPA\right)+ {f}_{2}\left(SB\right)+{f}_{3}\left(Sleep\right)+\varepsilon$, where ${f}_{1}$, ${f}_{2}$, ${f}_{3}$ are smooth functions
1E	CoDA isometric log-ratio model	$FPG= {\widehat{\zeta }}_{0}+{\widehat{\zeta }}_{1}\text{ln}\left(\frac{MVPA}{\sqrt[3]{SB\times Sleep\times LPA)}}\right)+ {\widehat{\zeta }}_{2}\text{ln}\left(\frac{SB}{\sqrt[2]{Sleep\times LPA)}}\right)+ {\widehat{\zeta }}_{3}\text{ln}\left(\frac{Sleep}{LPA}\right)+\varepsilon$
**Compositional data with variable totals (energy intake)**		
2A	Linear leave-one out model (classic “isocaloric model”)	$FPG= {\widehat{\eta }}_{0}+{\widehat{\eta }}_{1}Carbs+ {\widehat{\eta }}_{2}Fat+{\widehat{\eta }}_{3}Alcohol +{\widehat{\eta }}_{4}Total+\varepsilon$
2B	Ratio leave-one-out model (“nutrient density model”)	$FPG= {\widehat{\theta }}_{1}+{\widehat{\theta }}_{1}\frac{Carbs}{Total}+ {\widehat{\theta }}_{2}\frac{Fat}{Total}+{\widehat{\theta }}_{3}\frac{Alcohol}{Total}+\varepsilon$
2B'	Ratio leave-one-out model with total (“multivariable nutrient density model”)	$FPG= {\widehat{\iota }}_{0}+{\widehat{\iota }}_{1}\frac{Carbs}{Total}+ {\widehat{\iota }}_{2}\frac{Fat}{Total}+{\widehat{\iota }}_{3}\frac{Alcohol}{Total}+{\widehat{\iota }}_{4}Total+\varepsilon$
2C	Log2 leave-one-out model	$FPG= {\widehat{\kappa }}_{0}+{\widehat{\kappa }}_{1}{log}_{2}Carbs+ {\widehat{\kappa }}_{2}{log}_{2}Fat+{\widehat{\kappa }}_{3}{log}_{2}Alcohol +{\widehat{\kappa }}_{4}{log}_{2}Total+\varepsilon$
2C'	Log2 all-components model	$FPG= {\widehat{\lambda }}_{0}+{\widehat{\lambda }}_{1}{log}_{2}Carbs+ {\widehat{\lambda }}_{2}{log}_{2}Fat+{\widehat{\lambda }}_{3}{log}_{2}Alcohol +{\widehat{\lambda }}_{4}{log}_{2}Protein+\varepsilon$
2D	GAM leave-one-out model	$FPG= {\widehat{\mu }}_{0}+{g}_{1}\left(Carbs\right)+ {g}_{2}\left(Fat\right)+{g}_{3}\left(Alcohol \right)+{g}_{4}\left(Total\right)+\varepsilon$, where ${g}_{1}$, ${g}_{2}$, ${g}_{3}$ are smooth functions
2D'	GAM all-components model	$FPG= {\widehat{\nu }}_{0}+{h}_{1}\left(Carbs\right)+ {h}_{2}\left(Fat\right)+{h}_{3}\left(Alcohol \right)+{h}_{4}\left(Protein\right)+\varepsilon$, where ${h}_{1}$, ${h}_{2}$, ${h}_{3}$ are smooth functions
2E	CoDA isometric log-ratio model	$FPG= {\widehat{\xi }}_{0}+{\widehat{\xi }}_{1}\text{ln}\left(\frac{Carbs}{\sqrt[3]{Fat\times Alcohol\times Protein)}}\right)+ {\widehat{\xi }}_{2}\text{ln}\left(\frac{Fat}{\sqrt[2]{Alcohol\times Protein)}}\right)+{\widehat{\xi }}_{3}\text{ln}\left(\frac{Alcohol}{Protein}\right)+\varepsilon$

Models 1A and 2A, for physical activity and energy intake, respectively, represent the most straightforward and commonly used approach: simple linear ‘leave-one-out’ regression models which include the total and all components *except* the reference being substituted. In 1A the total is implicitly included since it is fixed, whereas in 2A the total varies and has to be explicitly included. We refer to these analytical models as ‘linear’, to denote the fact that they do not include any non-linear parameterisations, even though the other models that follow are also linear models (in the transformed variables).

Models 1B and 2B are linear regression models in which the compositional components are represented as simple ratio variables, where the numerator component is the compositional total as the denominator (known as ‘nutrient density models’ in nutrition epidemiology). For total energy intake (i.e. where the total varies), we examine an additional variation of this model, 2B′, that also adjusts for the total (known as the ‘multivariable nutrient density model’). We refer to these analytical models as ‘ratio’ models for convenience since this is their main parametric feature, even though the isometric log-ratio models that follow are also strictly ratio models.

Models 1C and 2C are leave-one-out models that are similar to 1A and 2A, except each term is parametrised with a log_2_ transformation. Where the total varies (i.e. the total energy intake example), we also examine an ‘all-components’ variation of this model, 2C′, that contains the ‘left out’ component instead of the total [17].

Models 1D and 2D are simple non-parametric leave-one-out models known as generalized additive models. They are similar to 1C and 2C, respectively, but instead of assuming a specific parametric relationship (e.g. log_2_), each compositional component has its own smoothing function that theoretically allows any smooth relationship to be modelled. We also examine an all-components version of each model, that contains the ‘left out’ component instead of the total. The basis dimensions (k) for these models, which controls the complexity of the functions used, were selected for each scenario by comparing the Akaike information criterion (AIC) between a variety of potential models (with k = 5, 10, 15, 20, 25, or 30 for all terms).

Finally, models 1E and 2E model the outcome using isometric log-ratio pivot coordinates of the compositional components, commonly used in CoDA.

To obtain the effect estimates from each model, we predicted and compared the outcome under specific combinations of the compositional components. Specifically, we compared the prediction at the geometric mean for all components, for 1- and 10-min increases in MVPA at the expense of LPA, and for 1- and 100-kcal increases in carbohydrates at the expense of protein. These values were selected to represent realistic substitutions between the respective components, balancing both the specifics of the data (the range of values possible to extrapolate to) and the feasibility of substitutions in practice. For example, 10-kcal substitutions would have been too small to be practically meaningful, whereas 100-min physical activity substitutions might not have been practically achievable. Each of these is presented alongside the effect of a single-unit change, as is common in epidemiology. The geometric mean was chosen as the starting point for all reallocations because CoDA methods typically present estimates of changes from the geometric mean; this allows the performance of all models to be directly compared.

The full model building process and details on the calculation of specific substitution effects are available in the annotated Supplementary Code.

## Results

**Fig. 1:**
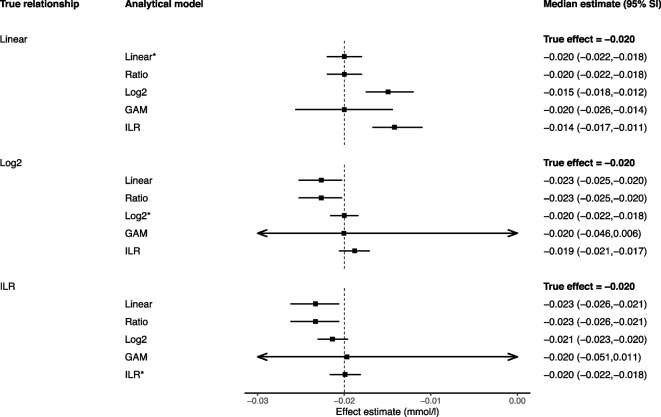
Performance of different models for estimating a 1-min reallocation in compositional data with fixed totals. Legend: *Data-generating model; GAM, generalized additive model; ILR, isometric log-ratio; SI, simulation intervals. The reported estimates represent the median and 95% simulation intervals from 10,000 simulated datasets for the effect of a 1-min increase in MVPA from 20.17 min to 21.17 min and a corresponding 1-min decrease in LPA from 308.61 min to 307.61 min

**Fig. 2:**
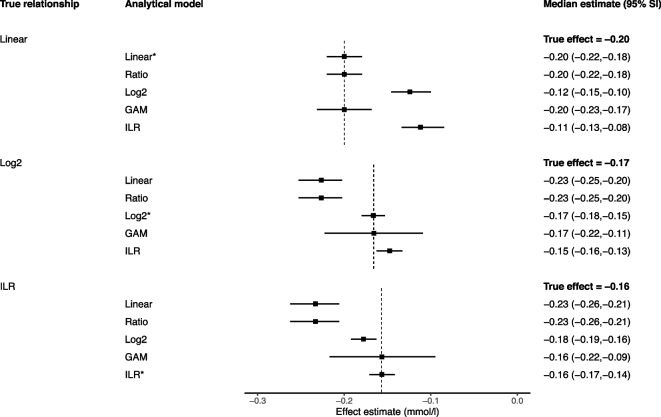
Performance of different models for estimating a 10-min reallocation in compositional data with fixed totals. Legend: *Data-generating model; GAM, generalized additive model; ILR, isometric log-ratio; SI, simulation intervals. The reported estimates represent the median and 95% simulation intervals from 10,000 simulated datasets for the effect of a 10-min increase in MVPA from 20.17 min to 30.17 min and a corresponding 10-min decrease in LPA from 308.61 min to 298.61 min

**Fig. 3:**
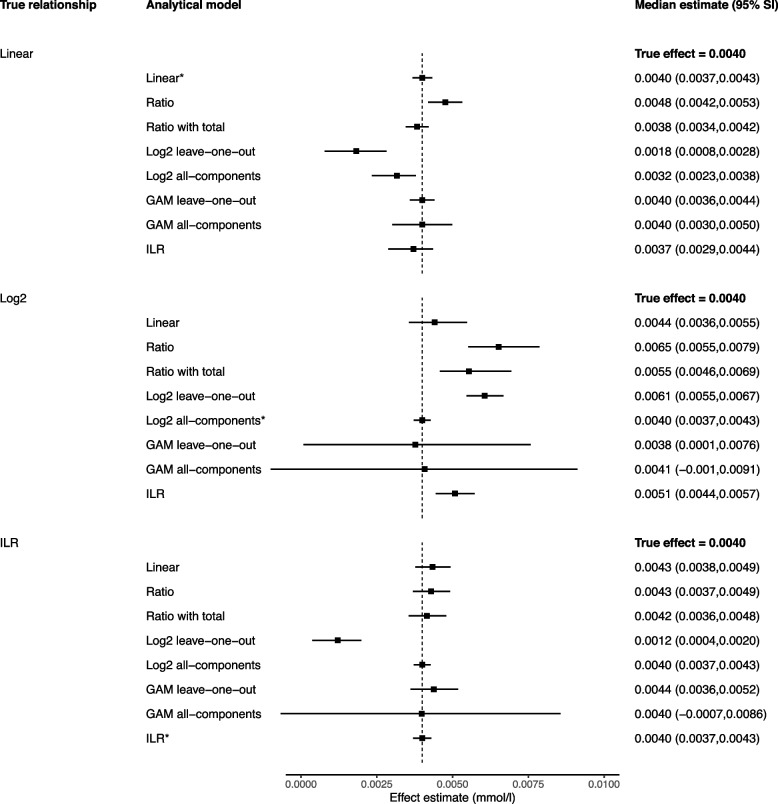
Performance of different models for estimating a 1-kcal reallocation in compositional data with variable totals. Legend: *Data-generating model; GAM, generalized additive model; ILR, isometric log-ratio; SI, simulation intervals. The reported estimates represent the median and 95% simulation intervals from 10,000 simulated datasets for the effect of a 1-kcal increase in carbohydrates from 927.12 kcal to 928.12 kcal and a corresponding 1-kcal decrease in protein from 280.28 kcal to 279.28 kcal

**Fig. 4:**
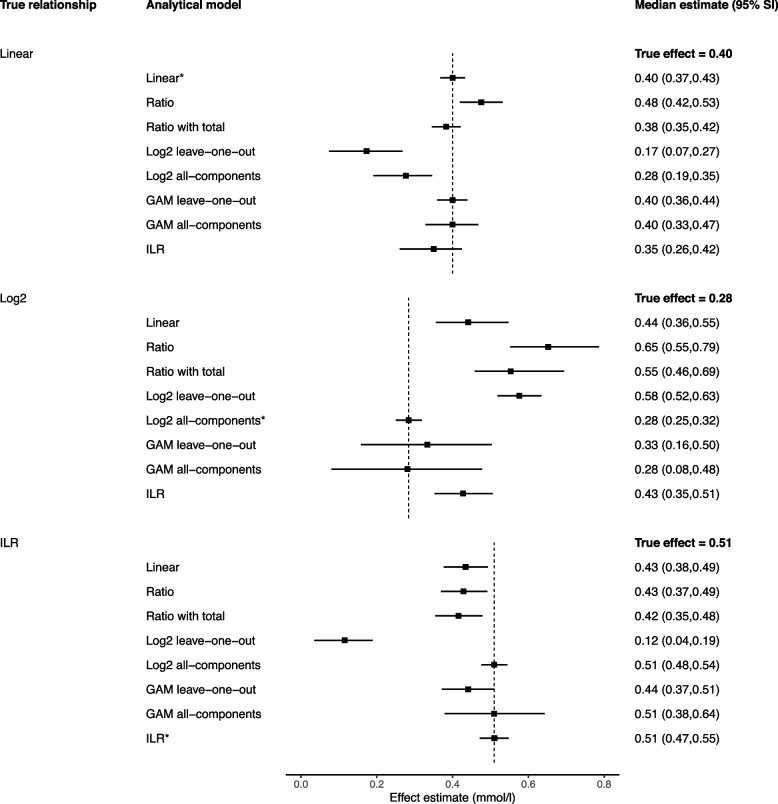
Performance of different models for estimating a 100-kcal reallocation in compositional data with variable totals. Legend: *Data-generating model; GAM, generalized additive model; ILR, isometric log-ratio; SI, simulation intervals. The reported estimates represent the median and 95% simulation intervals from 10,000 simulated datasets for the effect of a 100-kcal increase in carbohydrates from 927.12 kcal to 1027.12 kcal and a corresponding 100-kcal decrease in protein from 280.28 kcal to 180.28 kcal

The full results from each model in each scenario, including corresponding 95% simulation intervals (SIs) (the 2.5th and 97.5th effect estimate centiles from the 10,000 simulations) are presented in Figs. 1 and 2 (for physical activity compositional data with fixed totals) and Figs. 3 and 4 (for dietary compositional data with variable totals).

All presented reallocations refer to changes from the geometric mean, e.g. a 10-min reallocation between MVPA and LPA refers to a change from MVPA = 20.17 min and LPA = 308.61 min (the geometric means) to MVPA = 30.17 min and LPA = 298.61 min (10-min change from the geometric means in opposite directions). For all models with non-linear terms, the effects presented refer to reallocations from the geometric mean only; the effects would be different if the reallocation started from a different position.

### Linear leave-one-out models

When estimating 1-unit substitutions, the linear leave-one-out approach (model 1A, the classic isotemporal model, and model 2A, the classic isocaloric model) performed reasonably in both the fixed totals (Fig. 1) and variable totals settings (Fig. 2), regardless of the true data generating process. All estimates were identical to, or within 20% of, the truth. With larger substitutions, however, the linear leave-one-out model performed less well, with consistently larger bias in both types of compositional data whenever the true data generating mechanism was not linear (Figs. 3 and 4).

### Ratio variables models

In compositional data with fixed totals (i.e. physical activity data), the simple ratio model (1B) produced the same estimates as the linear leave-one-out-model, demonstrating they are mathematically equivalent in this context (Figs. 1 and 3). However, in compositional data with variable totals (i.e. dietary data), the results diverged (Figs. 2 and 4). Without adjustment, the ratio model returned biased estimates in all scenarios. The model performed best when the truth was isometric log-ratios, but a biased estimate was still obtained for the 100-kcal reallocation.

When additional adjustment was made for the variable total (2B′), the ratio model performed better when the true relationship was linear (e.g. 100-kcal estimate with adjustment for total = 0.38 mmol/l, truth = 0.400 mmol/l), but all other estimates were similarly biased to the simple ratio model.

### Non-linear leave-one-out and all-component models

The log_2_ leave-one-out model (1C and 2C) was very unreliable, only producing an accurate estimate in compositional data with fixed totals when the model matched the data generating mechanism (Figs. 1 and 3). Bias was otherwise present in all scenarios, especially in compositional data with variable totals (Figs. 2 and 4). This included the scenario when the true data generating mechanism was log_2_ (e.g. 100-kcal estimate = 0.58 mmol/l, truth = 0.28 mmol/l). The log_2_ all-components model (models 2C′) performed better, providing accurate estimates under both the log_2_ and isometric log-ratio scenarios.

### Generalised additive models

In compositional data with fixed totals, generalised additive models produced accurate average estimates in all scenarios albeit with very wide simulation intervals, suggesting low precision (an example of a bias-variance trade-off) (Fig. 1). However, the precision was substantially improved when estimating a 10-unit rather than 1-unit substitution (Fig. 3).

In compositional data with variable totals, the leave-one-out generalised additive models experienced bias in both non-linear scenarios (Fig. 2). This bias disappeared when all-components generalised additive models were used. Again, the model precision was substantially improved when estimating a 100-unit rather than 1-unit substitution (Fig. 4).

### Compositional Data Analysis (CoDA)

In compositional data with fixed totals, when estimating a 1-unit substitution, the CoDA model (1E) performed poorly in the linear scenario, but returned estimates that were identical to, or indistinguishable from, the truth in the log_2_ and isometric log-ratio scenarios (Fig. 1). However, for a 10-unit change, the approach only performed accurately when the truth was based on isometric log-ratios (Fig. 3).

When estimating 1-unit changes in compositional data with variable totals, the CoDA model (2E) returned accurate or reasonable estimates for both the isometric log-ratio and linear scenarios (Fig. 2). However, for a 100-unit change, it only returned an accurate estimate when the true relationship was based on isometric log-ratios (Fig. 4).

## Discussion

### Principal findings

This study examined the performance of the three main approaches to estimating the causal effects of compositional exposures: leave-one-out models, ratio variables models, and CoDA models. We examined both compositional data with fixed totals and compositional data with variable totals using the examples of physical activity and dietary data, respectively.

Our findings show that an accurate estimate can only be guaranteed when the model parameterisation matches the data generating process. Simple linear leave-one-out models offered reasonable estimates in all scenarios, provided the target substitution effect was small. However, for larger effects (i.e. 10-unit or 100-unit substitutions), the bias from an incorrectly parameterised model increased substantially. As expected, we also demonstrate that ratio variable models are equivalent to simple linear models with count variables when the compositional total is fixed (such as in physical activity) but not when the compositional total varies (such as in dietary data). In the context of variable totals, we show that leave-one-out models with non-linear terms do not return accurate causal effect estimates, even when using the correct non-linear parameterisations, but fully partitioned (all-components) models estimate these correctly. All-component generalized additive models returned unbiased estimates in all scenarios, but experienced considerable uncertainty (i.e. variability over simulations). However, this uncertainty was much smaller when estimating larger substitution effects, which may often be preferred (e.g. 1-kcal substitutions are unlikely to be of clinical interest, compared to 100-kcal ones).

### Appropriateness of leave-one-out models

One of the biggest criticisms regarding leave-one-out approaches, such as the isotemporal or isocaloric models, is that they are “non-compositional” [21, 34, 35]. This stems from two claimed features of such models: 1) all terms are included as counts, rather than using parameterisations that make the components inherently relative to each other, and 2) the linear model produces symmetrical estimates, where the effect of adding 1 unit is the same as subtracting 1 unit, which may be implausible. We believe this reasoning is fallacious, because it is not the *model* that is compositional but the *data.* As long as a suitable model is used, and the parameterisation matches the data generation process, then it will produce accurate effect estimates. Some may argue that only CoDA-informed models, such as those involving isometric (or other) log-ratios, are suitable for analyses of compositional data [11, 12]. However, in analyses involving outcomes that are not part of the same composition as the exposure variables (which is typically the case), it seems plausible that many alternative parameterisations—including linearity—may be reasonable or necessary.

Unfortunately, unless only small substitutions are of interest, the leave-one-out approach is unsuitable in compositional data with variable totals (such as dietary data) whenever non-linear parameterisations are necessary. We have previously shown that leave-one-out models are susceptible to residual confounding bias and ‘composite variable bias’ if the part ‘left out’ of the model represents more than one component, explicitly or otherwise. To avoid this, we suggested using an ‘all-components’ approach and deriving the estimates from the relevant coefficients [17]. This approach has previously been termed the ‘partition’ or ‘decomposition’ model, but we prefer the term ‘all-components’ as it clearly differentiates between the fully partitioned and other versions of the partition model. The leave-one-out and partition models are typically described as mathematically equivalent, provided the leave-one-out model is correctly specified (i.e. includes all-but-one term in the same units) [32]. However, this is only true when both models are linear. As we demonstrate, the leave-one-out model fails to provide accurate substitution effects in compositional data with variable totals when either the exposure or the substituting component has a non-linear relationship with the outcome. Since the ‘all-components’ model does not rely on the same additive structure, it can be used in these circumstances to model non-linear relationships. Of course, each term (particularly the exposure and substituting component) must still be parameterised correctly.

### The substitution size matters

Our results show a clear difference in model performance when estimating 1-unit substitutions vs 10- or 100-unit substitutions. While several approaches were able to return accurate or reasonably accurate estimates for modest reallocations, even when their parameterisation did not match the data generation process, larger biases were apparent when greater-unit reallocations were estimated. This was particularly evident in compositional data with variable totals (dietary data), where only the models with parameterisations that *exactly* matched the data generation process returned accurate estimates. This is because the degree of divergence between different parametric relationships, e.g. between a linear relationship and a log_2_ relationship, is strongly related to the range being examined. This has important implications for applied analyses of compositional data where it is common to estimate the effect of large interventions (e.g. 10- or 100-units), since the accuracy of such effects will be highly reliant on the accuracy of the parameterisation. On the other hand, we found that generalized additive models generally performed better when estimating the effect of larger interventions due to greatly improved precision, presumably because larger comparisons are less vulnerable to local misfit in the smoothing functions.

### Ratio variables revisited

CoDA approaches are often justified as a means to resolve the ‘spurious correlations’ that Pearson warned can occur between ratio variables and ‘closed form’ compositional data [36–38]. However, our findings demonstrate that no such spurious associations are introduced by ratio variables, provided the compositional total is a constant, such as physical activity data (or is conditioned on such as dietary data with adjustment for energy intake). On the contrary, in these circumstances the linear ratio model performs identically (or similar) to a linear leave-one-out model. This discrepancy may be due to a misunderstanding of Pearson’s original work, which describes an artefact that can only occur when the ratios have *variable* denominators. In compositional data with fixed totals (physical activity), dividing all components by the total is equivalent to dividing all variables by a constant. No ‘spurious correlation’ can be introduced by such a transformation, since it introduces no variation, it simply rescales all variables by the same factor. The same cannot be said in compositional data with variable totals (dietary data), since the variable denominator can cause considerable bias [13, 17, 39–41]. It is for this reason that the standard ratio model (without further adjustment for the total) performed so poorly in our simulations.

This specific misunderstanding of compositional data is particularly important because most of the examples of CoDA approaches in practice are in time-use epidemiology, where the totals are fixed (e.g. 24 h/day), or only vary due to measurement error but not nature (e.g., non-wear time for wearable devices), and this is the one example where ratio variables may actually return robust estimates.

### Recommendations

Investigators need to recognise when data are compositional, as is common in physical activity and dietary data. Such data require special consideration to accurately estimate and interpret causal effects, such as in the context of substitution analyses. Compositional data with fixed and variable totals are not the same and may require a different interpretation [6] and modelling strategy. In both settings, obtaining accurate estimates of relative effects also relies on the parametric relationship being correctly modelled.

Consequently, studies seeking to estimate causal effects in compositional data should carefully consider the relationships between the compositional components and the outcome of interest and parameterise them accordingly. This may be less important when interested in small substitutions (such as 1-min or 1-kcal) but becomes critical for estimating larger and more practically meaningful reallocations. The shape of the relationship between the exposure and outcome may be examined visually using flexible non-parametric methods (such as generalized additive models or locally weighted scatterplot smoothing) or statistically by comparing model fit (e.g. using the AIC) between candidate models with different parameterisations. Model fit should also be scrutinised by conducting standard model diagnostics, in particular examining the residuals for patterns or non-constant variance that may indicate mis-paramaterisation. If the focal relationship appears approximately linear, then either the leave-one-out or all-components approaches may be considered. If the focal relationship is non-linear then non-linear terms will need to be explored and incorporated. In the case of compositional data with variable totals, only the all-components model is compatible with non-linear terms.

If researchers believe that the underlying data generation process is based on isometric log-ratio relationships, then appropriate CoDA analyses may be considered. However, given the risk of bias when the true relationship does not match the CoDA parametrisation, we recommend very carefully examining the model fit and assumptions. Even when CoDA parameterisations match the true data generating mechanism, alternative and simpler non-linear parameterisations may suffice. In our simulation, the log_2_ all-components model returned unbiased estimates in the CoDA scenario in compositional data with variables totals. While this result should not be generalised, it demonstrates the benefit of considering a range of potential approaches. Alternatively, generalized additive models may be worth considering if seeking to estimate a large reallocation where a suitable parameterisation cannot be identified. Readers are reminded that in non-linear scenarios a substitution effect estimated from a specific starting point cannot be extrapolated to another starting position; where a different reallocation is of interest, with a different starting position, this needs to be explicitly estimated.

Although they offer no benefit over linear count models, ratio variable models may be considered for analyses of compositional data with fixed totals, such as physical activity data, provided non-linearities are examined and modelled accordingly. In compositional data with variable totals, such as dietary data, ratio variable models should be avoided due to the risk of severe bias [17, 39]. This may be reduced by adjusting for the total, but the resulting leave-one-out model is only appropriate when all effects are linear.

### Limitations

We used simulated data to explore and demonstrate the performance of the different approaches. Although the data were designed to be plausible, the effects we report should not be interpreted as real effects. Although we simulated means that closely match real data, the standard deviations have been narrowed to avoid simulating too many (impossible) negative values. We did not simulate confounding or measurement error, despite these being ubiquitous concerns in observational data. This was to avoid distracting from the main messages, however the contribution of confounding is very important to some of the modelling decisions, as we have demonstrated previously [17]. We restricted our study to examining three main parametrisations: linear, log_2_, and isometric log-ratios. Other parameterisations exist and may be useful in certain situations, both for CoDA approaches (e.g. centred log-ratios) and non-CoDA approaches (e.g. fractional polynomials). Similarly, although we simulated a range of data generating scenarios, the true data generating mechanism will vary in practice and will be unknown. To balance the number of models presented, we only included 1-, 10-, and 100-unit change substitutions as illustrative examples and did not consider further sensitivity analyses. It is possible, therefore, that the findings may be somewhat simplified and may not generalise to all other settings.

## Conclusions

Compositional data with fixed and variable totals behave differently. In addition to recognising how to interpret such data, the choice of modelling approach is also vital. Investigators must respect the parametric relationship between the components and the outcome and model it correctly. The larger the reallocation of interest, the more important this is. The implications of incorrectly parameterising a model may be more severe in compositional data with variable totals. As long as the model is carefully selected to correctly parameterise the relationship of interest, all existing approaches to analysing compositional data have utility.

## Supplementary Information

Supplementary material 1: Supplementary Code (variable totals). Supplementary Code (fixed totals).

Supplementary material 2. Table S1.

Supplementary material 3: Table S2.

## Data Availability

All data generated or analysed during this study are included in this published article and its supplementary information files. The code that simulates the data is available in Supplementary Code.

## Abbreviations

AIC: Akaike information criterion
CoDA: Compositional data analysis
FPG: Fasting plasma glucose
GAM: Generalised additive model
ILR: Isometric log-ratio
LPA: Light physical activity
MVPA: Moderate-to-vigorous physical activity
SB: Sedentary behaviour
