# Supplementary material for: A comparison of methods for analysing compositional data with fixed and variable totals: a simulation study using the examples of time-use and dietary data
Source: BMC Med Res Methodol. 2025 Apr 17;25:100. doi: 10.1186/s12874-025-02509-1 (PMC12004694; doi:10.1186/s12874-025-02509-1)
Supplement: Supplementary file 2 — Supplementary material 2. Table S1. [file 12874_2025_2509_MOESM2_ESM.docx]

**Supplementary Table 1.** Average of the means and standard deviations of the variables created in the simulations.

| **Variable** | **Mean** | **SD** |
| --- | --- | --- |
|  |  |  |
| **Compositional data with a fixed total (physical activity)** | |  |
|  | |  |
| MVPA (mins) | 22 | 7.9 |
| Sedentary behaviour (mins) | 541 | 42.9 |
| Sleep (mins) | 564 | 49.9 |
| LPA (mins) | 313 | 51.8 |
| FPG (created with linear model) | 5.5 | 0.8 |
| FPG (created with Log2 model) | 5.5 | 1.2 |
| FPG (created with ILR model) | 5.5 | 1.9 |
| MVPA (% of total time) | 1.5 | 0.6 |
| Sleep (% of total time) | 39.2 | 3.5 |
| Sedentary behaviour (% of total time) | 37.6 | 3.0 |
| LPDA (% of total time) | 21.7 | 3.6 |
|  |  |  |
| **Compositional data with variable totals (energy intake)** |  |  |
|  |  |  |
| Carbohydrates (calories) | 1000 | 343 |
| Fat (calories) | 600 | 197 |
| Protein (calories) | 300 | 98.7 |
| Alcohol (calories) | 100 | 10 |
| Total (calories) | 2000 | 408 |
| FPG (created with linear model) | 5.5 | 1.8 |
| FPG (created with Log2 model) | 5.5 | 3.1 |
| FPG (created with ILR model) | 5.5 | 1.8 |
| Carbohydrates (% of total calories) | 49 | 11.1 |
| Fat (% of total calories) | 30.3 | 9.4 |
| Protein (% of total calories) | 15.4 | 5.6 |
| Alcohol (% of total calories) | 5.2 | 1.3 |
