# Supplementary material for: A comparison of methods for analysing compositional data with fixed and variable totals: a simulation study using the examples of time-use and dietary data
Source: BMC Med Res Methodol. 2025 Apr 17;25:100. doi: 10.1186/s12874-025-02509-1 (PMC12004694; doi:10.1186/s12874-025-02509-1)
Supplement: Supplementary file 3 — Supplementary material 3: Table S2. [file 12874_2025_2509_MOESM3_ESM.docx]

**Supplementary Table 2.** Values of each parameter used in the data generating models shown in Table 1

| **True model parameter** | **Value** |
| --- | --- |
| **Fixed totals –Simple linear model** |  |
| $\alpha_{0}$ | 0.621902 |
| $\alpha_{1}$ | -0.02 |
| $\alpha_{2}$ | 0.015 |
| $\alpha_{3}$ | -0.005 |
| **Fixed totals – Log_2_ model** |  |
| $\gamma_{0}$ | -41.1904 |
| $\gamma_{1}$ | -0.286545 |
| $\gamma_{2}$ | 8.12 |
| $\gamma_{3}$ | -2.82 |
| **Fixed totals – Isometric log-ratio model** |  |
| $\zeta_{0}$ | 3.4 |
| $\zeta_{1}$ | -0.5196152 |
| $\zeta_{2}$ | 14.0359 |
| $\zeta_{3}$ | -5.261348 |
| **Fixed totals – noise term** |  |
| $\sigma_{1}$ | 0.25 |
| **Variable totals – Simple linear model** |  |
| $\eta_{0}$ | 0.2 |
| $\eta_{1}$ | 0.005 |
| $\eta_{2}$ | -0.001 |
| $\eta_{3}$ | 0.001 |
| $\eta_{4}$ | 0.006 |
| **Variable totals – Log_2_ model** |  |
| $\lambda_{0}$ | -45 |
| $\lambda_{1}$ | 4.73678 |
| $\lambda_{2}$ | -0.6 |
| $\lambda_{3}$ | 0.652776 |
| $\lambda_{4}$ | 0.6 |
| **Variable totals – Isometric log-ratio model** |  |
| $\xi_{0}$ | 1 |
| $\xi_{1}$ | -0.5196152 |
| $\xi_{2}$ | 3.865826 |
| $\xi_{3}$ | -0.4839213 |
| **Variable totals – noise term** |  |
| $\sigma_{2}$ | 0.5 |
